# Relationship between fluoroquinolones and the risk of aortic diseases: a meta-analysis of observational studies

**DOI:** 10.1186/s12872-020-01354-y

**Published:** 2020-02-03

**Authors:** Xiao-ce Dai, Xin-xin Yang, Lan Ma, Guan-min Tang, Yan-yun Pan, Hui-lin Hu

**Affiliations:** 1 Department of Cardiology, Affiliated Hospital of Jiaxing University, No.1882 Zhonghuan South Road, Jiaxing, Zhejiang, China; 2 Department of Cardiology, First Affiliated Hospital of Zhejiang Chinese Medical University, Hangzhou, China; 3 Department of Traditional Chinese Medicine, Nantong No.1 People Hospital, Nantong, China

**Keywords:** Fluoroquinolones, Aortic aneurysm, Aortic dissection, Meta-analysis

## Abstract

**Background:**

Our aim was to determine the relationship between the use of fluoroquinolones and the risk of aortic diseases.

**Methods:**

PubMed, EMBASE and the Web of Science were searched from inception to July 6, 2019, to identify observational studies that evaluated the risk of aortic diseases associated in users of fluoroquinolones compared with nonusers or users of other antibiotics. The primary outcome was the first occurrence of aortic diseases. We used the GRADE approach to rate the strength of evidence. We used the inverse variance method random-effect model to estimate the odds ratios (ORs) with 95% CIs, and statistical heterogeneity was assessed by the *I*^*2*^ statistic.

**Results:**

This meta-analysis enrolled 2,829,385 patients reported the relationship between fluoroquinolones and the risk of aortic diseases. Compared with nonusers or users of other antibiotics, users of fluoroquinolone had a significantly increased risk of aortic diseases (adjusted OR, 2.10; 95% CI, 1.65–2.68; *P* = .000, *I*^*2*^ = 16.4%). The quality of evidence was moderate, and the number needed to harm (NNH) for aortic diseases among patients was estimated to be 1301.

**Conclusions:**

The fluoroquinolone use in patients significantly increases the risk of new-onset aortic diseases. Clinicians need to pay attention to these severe adverse events when considering fluoroquinolone use.

## Background

Fluoroquinolones are a group of broad-spectrum antibiotics that can effectively kill or stop the growth of gram-negative and gram-positive bacteria [1]. For this reason, they are routinely prescribed by internists, family practitioners, specialists, subspecialists and surgeons [2]. However, several studies have reported serious adverse events, such as aortic aneurysm and aortic dissection, [3, 4] in patients using fluoroquinolones. Aortic aneurysm and aortic dissection have high complication rates and carry a high risk of mortality, [5, 6] so the FDA announced the addition of these severe side effects to the warning about fluoroquinolones on December 20, 2018. To investigate these rare but serious events, two meta-analyses evaluated the relationship between the use of fluoroquinolones and the risk of aortopathy, [2, 7] but both articles had some limitations, such as the quantity of included studies. Recently, the European Commission has restricted the use of fluoroquinolones due to the possibility of permanent adverse effects [8]. An informed decision about fluoroquinolone use is needed for individuals to determine whether it is worth taking the risk. Hence, it is important for both health care providers and patients to be aware of the risks and benefits of fluoroquinolone use. Therefore, it is necessary to expand the research population to render the conclusions of a meta-analysis more reliable.

Based on previous meta-analyses, our aim was to reevaluate the effect of fluoroquinolone use on the risk of aortic diseases (aortic aneurysm and aortic dissection).

## Methods

This meta-analysis adhered to the Meta-analysis of Observational Studies in Epidemiology (MOOSE) [9] and Preferred Reporting Items for Systematic Reviews and Meta-Analyses (PRISMA) [10] guidelines. The protocol was registered with PROSPERO, and the number was CRD42019129056.

### Literature search

Systematic electronic searches in PubMed, EMBASE and Web of Science were performed from the date of database inception to March 2019. Electronic searches were conducted with exploded Medical Subject Headings (MeSH) terms and related key words. The search terms used were (MeSH exp. “aorta” and key word “aorta”) and (MeSH exp. “dissection” and key word “dissection”) and (MeSH exp. “aortic aneurysm” and key words “aortic aneurysm” and “aneurysm”) and (key word “fluoroquinolone*”). The detailed search strategy for each of the databases was provided in the PROSPERO protocol. We did not impose language restrictions. To ensure that we obtained the maximum number of studies possible, we reran the search on July 6, 2019.

### Selection criteria

Two authors (X.-C. D. and X.-X. Y.) independently conducted the original search, removed duplicate articles, read the relevant titles and abstracts and classified records as included, excluded or uncertain. In cases of ambiguity, the full-text article was read to clarify its eligibility. Discussion and consensus were used to resolve discrepancies.

Published cohort or case-control studies that met the following criteria were included:

1. Participants: Participants were adults not younger than 18 years who were treated for any condition with any fluoroquinolone.
2. Exposures: Patients received any of the following fluoroquinolones: ciprofloxacin, levofloxacin, enoxacin, sparfloxacin, norfloxacin, lomefloxacin, moxifloxacin, pefloxacin, ofloxacin, besifloxacin, gatifloxacin and gemifloxacin.
3. Control: The controls included either patients who did not receive any antibiotics or those exposed to other antibiotics.
4. Outcomes: The major outcome of interest was the first occurrence of aortic diseases.
5. Data: Studies reported the unadjusted or adjusted relative risk (RR), hazard ratio (HR), odds ratio (OR) or risk difference (RD) and the associated confidence intervals (CIs) or provided sufficient data to estimate these parameters.

### Data extraction

Data extraction was performed by X.-C. D. and checked separately by other authors (X.-X. Y. and L. M.). The collected data included the following: first author, year of publication, participants, interventions, primary outcomes, study design, controls, covariate adjustment, exposure category, crude and adjusted RR, HR or OR and the associated 95% CIs. Extracted data were collected in a Word (Microsoft Corporation) file. The coauthors resolved discrepancies by discussion. Different follow-up durations and durations of fluoroquinolone use existed in the included studies. Although all follow-up data were recorded, we chose a 60-day period from the start of treatment as the follow-up end point because that yielded the highest RRs, HRs or ORs.

### Risk of Bias assessment

Two authors (X.-C. D. and G.-M. T.) independently assessed the risk of bias using the Newcastle Ottawa Scale (NOS) for cohort and case-control studies [11]. The NOS rates case-control studies based on the case definition, representativeness of cases, selection and definition of controls, comparability of controls, ascertainment of exposure, use of the same method for the ascertainment of cases and controls and nonresponse rate. The NOS rates cohort studies based on the representativeness of the exposed cohort, representativeness of the nonexposed cohort, ascertainment of exposure, absence of the outcome of interest at the beginning of study, comparability of cohorts because of the design or analysis, assessment of outcome, follow-up long enough for outcomes to occur and adequacy of follow-up of cohorts. Studies were categorized as low quality (fewer than 5 stars), moderate quality (5–7 stars) and high quality (more than 7 stars).

### Accessing the quality of evidence

The quality of the evidence was classified as very low, low, moderate, or high for the primary outcome according to the Grading of Recommendations Assessment, Development, and Evaluation (GRADE) [12] methodology for the risk of bias, inconsistency, indirectness, and imprecision, and publication bias was independently evaluated by two authors (X.-C. D. and Y.-Y. P.). The GRADE Profiler (Windows-only tool GRADEpro) was used to construct summary tables.

### Statistical analysis

A random effects model with the inverse variance method was used to pool studies’ adjusted RRs, HRs and ORs, and we presumed that the OR was representative of the other measures, such as RR or HR, because aortic disease is rare event [13, 14]. Therefore, the OR, RR or HR based on propensity score matching or adjustment was used whenever available or was obtained from the adjusted multivariate analysis when propensity score matching had not been performed. We calculated ORs with 95% CIs for the overall effect estimate. Heterogeneity across studies was quantified using the *I*^*2*^ statistic; *I*^*2*^ > 50% was considered to indicate considerable heterogeneity [15]. *P* < .05 was considered statistically significant for all included analyses, except when otherwise specified. All statistical analyses were performed in STATA (Stata Version 14.0; Stata Corporation, College Station, TX, USA).

We calculated the number needed to harm (NNH) [16] and the corresponding 95% CI to estimate an absolute measure of effect because the OR is a relative measure of effect. The NNH is the number of patients who needed to be treated with fluoroquinolones for one additional patient to experience an adverse event. We used the pooled ORs to calculate the NNH using Rx software [17]. The baseline risk was acquired from the no antibiotics group or the group exposed to other antibiotics in the population-based studies.

## Results

### Trial selection

**Fig. 1:**
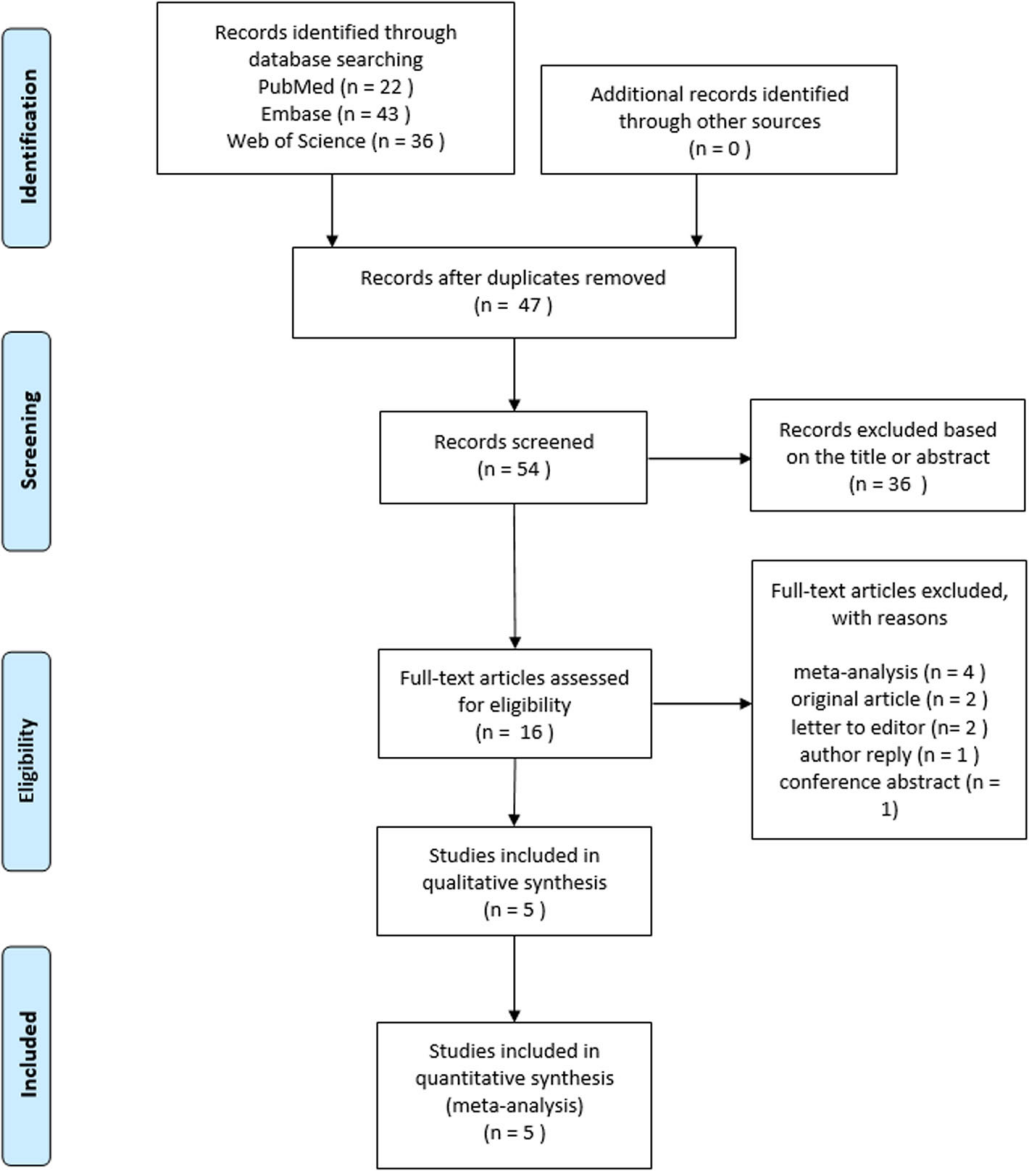
Selection of observational studies for the meta-analysis

The PRISMA study flowchart is shown in Fig. 1. Our initial search yielded 101 articles. A total of 16 records were thought to be preliminarily eligible for inclusion after screening the titles and abstracts and removing duplicates. After reviewing the full-text articles, our meta-analysis finally included 5 observational studies [3, 4, 18–20]. No randomized controlled trials were included because the incidence of aortic diseases is low and cannot be captured in randomized controlled trials with adequate power [21, 22].

### Trials characteristics

The study design and main characteristics of the included studies are summarized in (Additional file 1: Table S1). The publication years of the studies were between 2015 and 2019. The group sizes ranged from 2426 to 1,744,360, with 2,829,385 patients in total. All included studies were observational in nature, including two cohort studies, [19, 20] one nested case-control study, [18] one case-crossover design study [4] and one case/no case study [3]. The follow-up ranged from 60 days to 17 years; one article [3] did not report the follow-up duration. All studies adjusted the RRs, HRs or ORs, including 3 trials that used propensity score matching, [18–20] 1 trial that used a conditional logistic regression model [4] and 1 trial that used multivariable logistic regression [3]. All studies evaluated different fluoroquinolones, and the trials used a combination of administrative diagnostic codes to identify new-onset aortic diseases.

### Risk of Bias assessment

We summarized the detailed information about selection, comparability and exposure in the five trials, and the risk of bias according to the NOS assessment is shown in (Additional file 2: Table S2). Overall, the five studies were considered to be at low risk for bias, which meant that they were of high quality (8 stars for each study). Publication bias was assessed by funnel plots and the Egger and Begg test for asymmetry when it was available.

### Users of Fluoroquinolones vs. nonusers or users of other antibiotics

#### Primary outcome: the first occurrence of aortic dissection or aortic aneurysm

**Fig. 2:**
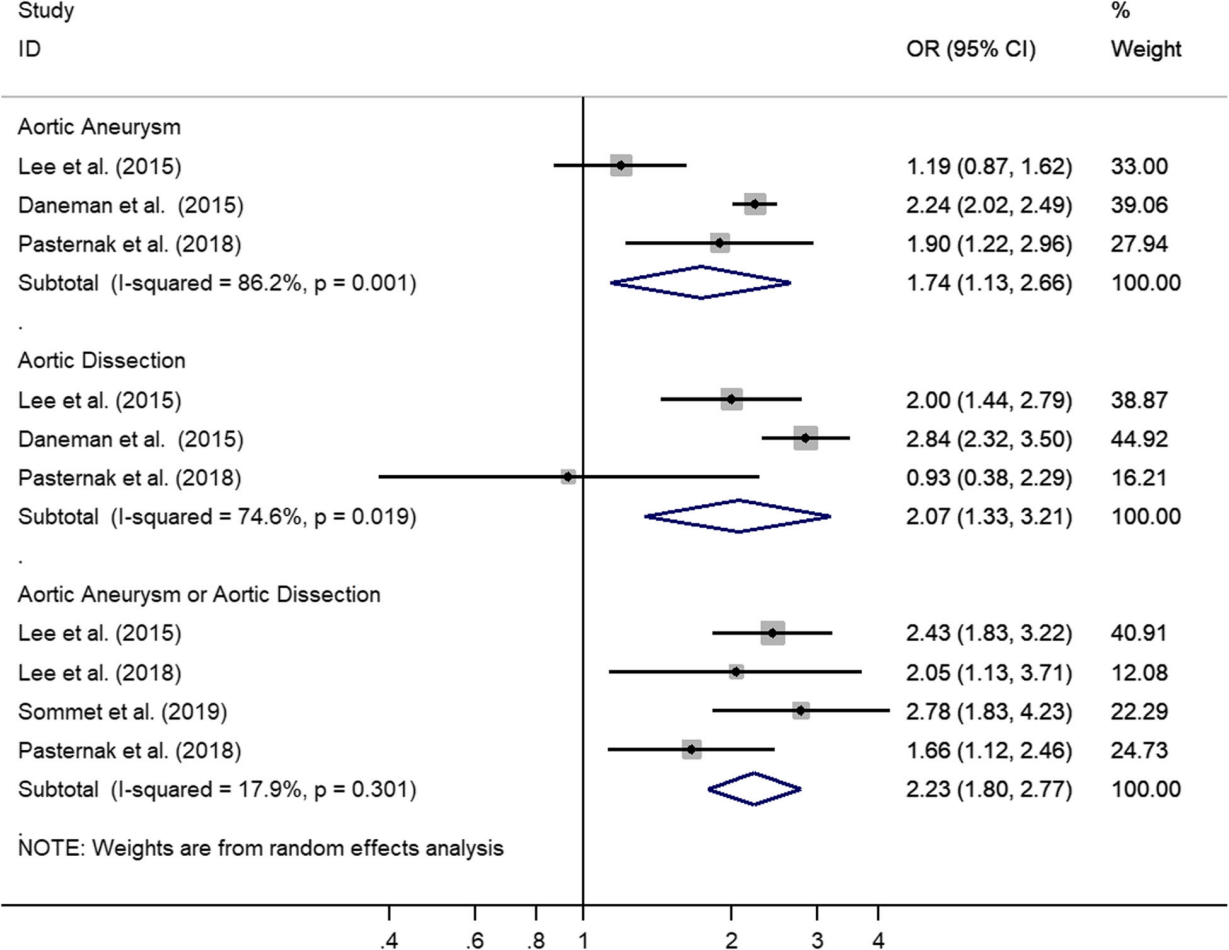
The Risk of Aortic Diseases among Fluoroquinolone Users in Comparison to Nonusers or Users of Other Antibiotics

Four trials provided data on the first occurrence of aortic diseases [3, 4, 18, 20]. Compared with nonusers or users of other antibiotics, users of fluoroquinolones had a substantially higher risk of aortic diseases (adjusted OR, 2.23; 95% CI, 1.80–2.77; *P* = .000) (Fig. 2), with low heterogeneity (*I*^*2*^ = 17.9%).

#### The first occurrence of aortic dissection and aortic aneurysm

Compared with nonusers or users of other antibiotics, users of fluoroquinolones also had a higher risk of aortic dissection (adjusted OR, 2.07; 95% CI, 1.33–3.21; *P* = .001), with very a high degree of statistical heterogeneity (*I*^*2*^ = 74.6%), and a higher risk of aortic aneurysm (adjusted OR, 1.74; 95% CI, 1.13–2.66; *P* = .011) (Fig. 2), also with a very high degree of statistical heterogeneity (*I*^*2*^ = 86.2%).

#### The NNH for aortic aneurysm or dissection

The NNH for aortic aneurysm or dissection with current fluoroquinolone use was 1301 (95% CI 852–2201) treatment courses of fluoroquinolones. One study with no follow-up was excluded, the effect estimate from our meta-analysis (adjusted OR = 2.10) was used, and a baseline risk (0.7/1000 person-years) was obtained from a Swedish nationwide cohort [20].

#### Sensitivity analysis

**Fig. 3:**
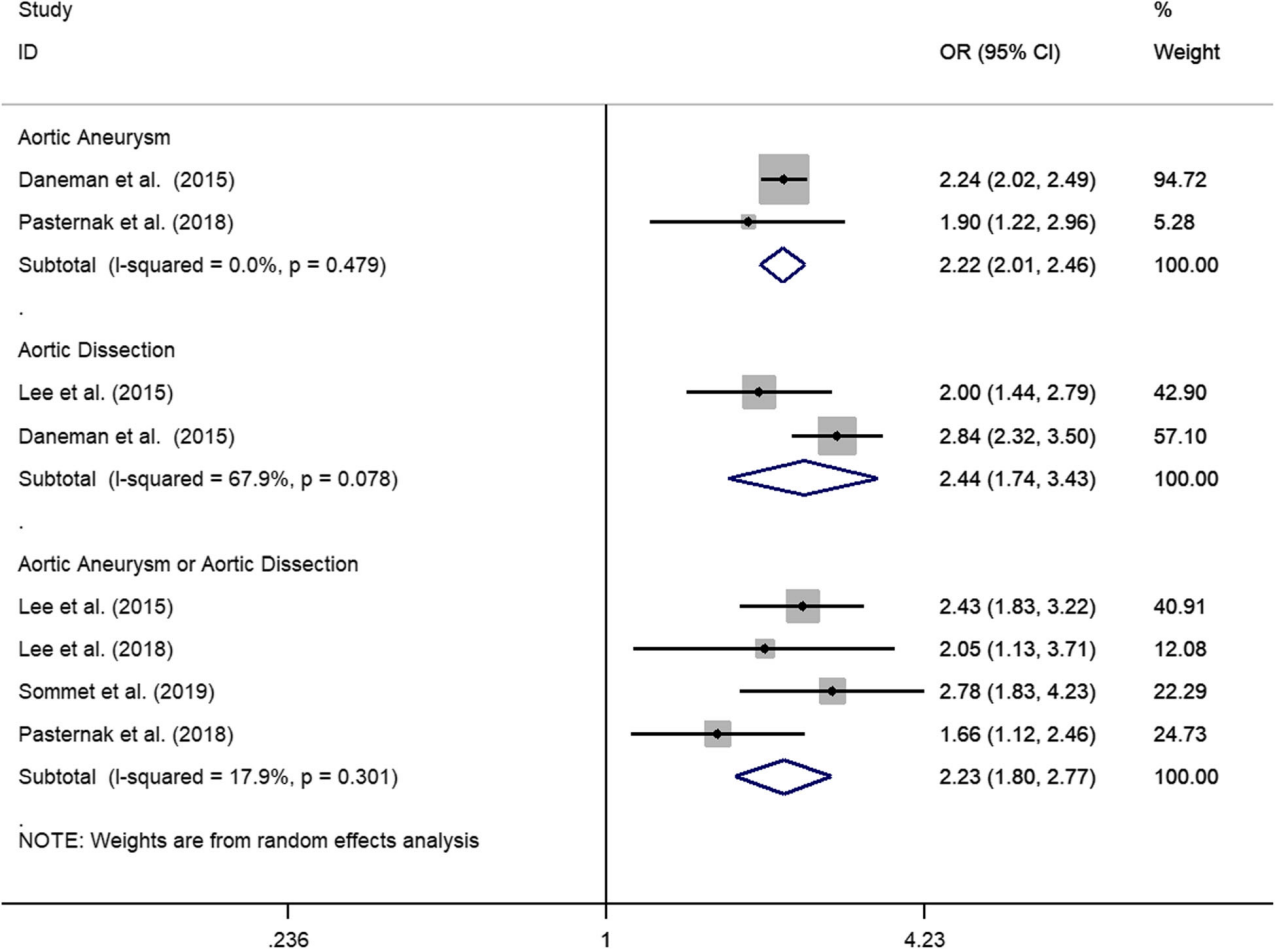
The Sensitive Analysis for the Risk of Aortic Diseases

The heterogeneity among studies reporting on aortic aneurysms changed to 0 after eliminating the study by Lee et al. [18] (adjusted OR, 2.22; 95% CI, 2.01–2.46; *P* = .000). The heterogeneity index ranged from 82.8 to 67.9% after excluding the study by Pasternak et al. [20] (adjusted OR, 2.44; 95% CI, 1.73–3.43; *P* = .000) (Fig. 3).

#### GRADE evidence and publication Bias

The GRADE level of evidence was moderate for the first occurrence of aortic diseases (fluoroquinolone users vs. nonusers or users of other antibiotics) because of the study design (observational study) and other considerations (large weight of the effect, no dose response gradient and no plausible confounding).

In this meta-analysis of the first occurrence of aortic dissection or aneurysm, we did not use tests such as Egger’s test of Begg’s test to assess publication bias, because the number of trials was low.

## Discussion

### Main findings

Our meta-analysis systematically reviewed the presently accessible literature and found that (1) users of fluoroquinolones in contrast to nonusers or users of other antibiotics had a significantly higher risk of aortic diseases and (2) despite current fluoroquinolone use doubling the risk of the occurrence of aortic dissection or aneurysm, the NNH was 1301 based on a moderate level of evidence.

### Comparison with other meta-analyses

Four meta-analyses on this issue have been published, and we have summarized the details in Table 1 [2, 7, 23, 24]. Though the major result of our meta-analysis was consistent with that of these former meta-analyses, differences and new findings between our meta-analysis and the former studies should be noted. First, these meta-analyses included no more than five studies. In contrast, our meta-analysis included 5 observational studies with 2,829,385 patients in total. Therefore, our meta-analysis is the most up-to-date and the broadest analysis; our analysis consolidates the results of the former meta-analyses. Second, we found that three of these meta-analyses used the HR of aortic aneurysm to replace the HR of aortic diseases [7, 23, 24]; this may underestimate the risk of occurrence of aortic diseases. We excluded this study, [19] when pooling the ORs. Third, we performed a sensitivity analysis; when we omitted the study by Lee et al. [18] from the analysis of aortic aneurysms, the heterogeneity index decreased to null. The risk of aortic disease was higher than the previous results because the OR in Lee’s study showed the opposite effect (OR = 0.87) from that observed in the other two studies. In the aortic dissection group, we eliminated the study by Pasternak et al. [20] and found that the high level of heterogeneity changed from 82.8 to 67.9%. These changes in estimates did not alter the statistical or clinical significance of the former findings. Therefore, we suggest that the result was robust. Moreover, we reevaluated the NNH for aortic aneurysm or dissection because we also found that the study by Lee et al. [18] had a more statistically significant RR than the pooled results in the two previous meta-analyses [7, 24]. Finally, after adjusting the data, we revaluated the quality of the evidence for the primary outcome to help medical care providers make clinical decisions.

**Table 1:** Comparison with Other Previous Meta-analyses

Author/Year	Singh S et al. 2017	Noman AT et al. 2019	Yu X et al. 2019	Rawla P et al. 2019	Current Meta-analysis
Number of observational studies	2	3	3	4	5
Number of participants	1,893,537	2,613,713	2,613,713	2,616,139	2,829,385
Year of search strategy	2017	2018	2019	2018	2019
OR/RR of AA or AD	AA (OR, 2.25; 95% CI,2.03–2.49)	AA or AD (OR,2.04; 95% CI, 1.67–2.48)	AA or AD (OR,2.20; 95% CI, 1.92–2.52)	AA or AD (RR,2.14; 95% CI, 1.93–2.36)	AA or AD (OR, 2.23; 95% CI, 1.80–2.77)
					AD (OR, 2.07; 95% CI, 1.33–3.21)
	AD (OR, 2.79; 95% CI,2.31–3.37)	AD (OR, 2.25; 95% CI,1.42–3.56)			
					AA (OR, 1.74; 95% CI, 1.13–2.66)
GRADE	moderate	moderate	NR	moderate	moderate
NNH	618 (AA)	1376	NR	NR	1301

*AD* aortic dissection, *AA* aortic aneurysm, *GRADE* Grading of Recommendations Assessment, Development, and Evaluation; *NNH* Number Need to Harm, *NR* not report

### Implications for clinical practice

This meta-analysis demonstrated the underlying risk of the first occurrence of aortic diseases among the older population above the age of 50 when these patients are exposed to fluoroquinolones. Moreover, when doctors weigh these severe side effects against the beneficial outcomes, the NNH for aortic disease should be taken into consideration. Therefore, for health care providers, if older patients have to use fluoroquinolones because the benefits outweigh the potential risks, regular follow-up during the first 60 days will be important.

There are multiple risks associated with fluoroquinolone use. Several studies showed that ciprofloxacin could inhibit collagen production in tenocytes [25, 26]. Another study suggested that patients with aortic aneurysm, aortic dissection or Marfan syndrome had a higher risk for aortic rupture when exposed to fluoroquinolones [27]. Moreover, compared with intravenous fluoroquinolones, oral fluoroquinolones carried a higher risk of negative effects, and levofloxacin was associated with not only aortic aneurysm but also aortic dissection [28]. Based on these findings, future studies should focus on age stratification, the evaluation of individuals’ fluoroquinolone with regard to the risk of aortic aneurysm or dissection and different exposure times, which may provide additional information because aortic diseases are rare adverse events that cannot be studied in randomized controlled trials.

### Strengths and limitations

An important strength of our meta-analysis was conformity with the PRISMA and MOOSE guidelines and the GRADE recommendations of the Cochrane Collaboration; we also registered the protocol with PROSPERO. To reevaluate the NNH for current fluoroquinolone use, we pooled the statistically significant ORs among studies and excluded one study that reported aortic aneurysm and aortic dissection separately.

Our meta-analysis had some limitations as well. First, the involved studies were all observational in design and were conducted with various patient groups, clinical settings and statistical methods. Second, the average age in the included studies was above 50 years, and patients from 18 to 49 years old were omitted. Third, the risk for aortic diseases in based on past use of fluoroquinolones was unclear because the result had no significant difference. Fourth, the populations might have had collagen disorders, inflammation or infection, and these conditions are also risk factors for the development of aortic wall weakness; the five included studies did not clarify whether fluoroquinolone use was the only risk factor. Fifth, the timing of fluoroquinolone intake was not mentioned in these five studies, so we cannot establish a temporal link between fluoroquinolone intake and the development of aortic diseases. Sixth, due to a lack of pharmacological interaction comparisons, we cannot determine whether fluoroquinolone use combined with other drugs, such as corticosteroids, increases the occurrence of aortic diseases. Last, some of these studies did not report each fluoroquinolone compared with all other fluoroquinolones, and the follow-up duration was not consistent across studies. We also did not perform meta-regression or an analysis of publication bias because of the absence of individual data and the limited number of studies.

## Conclusions

Our meta-analysis suggests that fluoroquinolone use results in a major increase in the new onset of aortic diseases, although the evidence quality was moderate and the absolute risk was fairly modest. Hence, physicians and surgeons should pay attention to the risk of aortic disease in patients taking fluoroquinolone.

## Supplementary information

**Additional file 1.** The Characteristics of Included Observational Studies Comparing Fluoroquinolone Use with Nonuse or Exposure to Other Antibiotics Regarding the Risk for Aortic Disease.

**Additional file 2.** Quality Assessment of Included Studies by Newcastle–Ottawa Scales.

## Data Availability

All data generated or analysed during this study are included in this published article.

## Abbreviations

CIs: Confidence intervals
GRADE: Grading of recommendations assessment, development, and evaluation
HR: Hazard ratio
MeSH: Medical subject headings
MOOSE: Meta-analysis of observational studies in epidemiology
NNH: Number needed to harm
NOS: Newcastle Ottawa scale
ORs: Odds ratios
PRISMA: Preferred reporting items for systematic reviews and meta-analyses
RD: Risk difference
RR: Relative risk
