# Supplementary material for: Relationship between fluoroquinolones and the risk of aortic diseases: a meta-analysis of observational studies
Source: BMC Cardiovasc Disord. 2020 Feb 3;20:49. doi: 10.1186/s12872-020-01354-y (PMC6998374; doi:10.1186/s12872-020-01354-y)
Supplement: Supplementary file 1 — Additional file 1. The Characteristics of Included Observational Studies Comparing Fluoroquinolone Use with Nonuse or Exposure to Other Antibiotics Regarding the Risk for Aortic Disease. [file 12872_2020_1354_MOESM1_ESM.docx]

**Table 1A** Characteristics of Included Observational Studies Comparing Fluoroquinolone Use With Nonuse or Exposure to Other Antibiotics Regarding the risk for Aortic Disease

| Study  Authors  Year | Participants | Interventions | Primary  Outcomes | Study design | Control | Follow-up | Covariate  adjustment | Exposure category | Outcomes | Adjusted RR (95% CI) |
| --- | --- | --- | --- | --- | --- | --- | --- | --- | --- | --- |
| Lee et al. 2015 | Adults in the National  Health Insurance Research Database (NHIRD) of Taiwan from 1998 to Dec 2011. | Use any of fluoroquinolones: ciprofloxacin, levofloxacin,  ofloxacin, sparfloxacin, norfloxacin, lomefloxacin, moxifloxacin,  gemifloxacin, enoxacin, pefloxacin | 1477 individuals with new-onset aortic  aneurysm(AA) or dissection (AD) requiring hospitalization | A nested case-control analysis | 147 700 controls using a risk set sampling scheme in the same database | 3613.3 days | Demographics, cardiovascular  comorbidities, risk factors for aortic aneurysm and  dissection, intensity of health care facility utilization, and use of specific medications, indication for fluoroquinolone use and a combined weighted comorbidity index. | Current use;  Within 60 days of the index date | AA or AD | 2.43 (1.83-3.22) |
|  |  |  |  |  |  |  |  |  | AA | 2.36 (1.66-3.36 |
|  |  |  |  |  |  |  |  |  | AD | 2.55 (1.58-4.11) |
|  |  |  |  |  |  |  |  | Past use;  61-365 days prior to the  index date | AA or AD | 1.48 (1.18-1.86) |
|  |  |  |  |  |  |  |  |  | AA | 1.19 (0.87-1.62) |
|  |  |  |  |  |  |  |  |  | AD | 2.00 (1.44-2.79) |
|  |  |  |  |  |  |  |  | Any prior-year use;  in the 1-year  period before the index date | AA or AD | 1.74 (1.44-2.09) |
|  |  |  |  |  |  |  |  |  | AA | 1.52 (1.19-1.94) |
|  |  |  |  |  |  |  |  |  | AD | 2.15 (1.61-2.85) |

**Table 1B** Characteristics of Included Observational Studies Comparing Fluoroquinolone Use With Nonuse or Exposure to Other Antibiotics Regarding the Risk for Aortic Disease

| Study  Authors  Year | Participants | Interventions | Primary  Outcomes | Study design | Control | Follow-up | Covariate  adjustment | Exposure category | Outcomes | Adjusted HR (95% CI) |
| --- | --- | --- | --- | --- | --- | --- | --- | --- | --- | --- |
| Daneman et al. 2015 | An inception cohort with uniform accrual of all  Ontario adults turning age 65, during a 15-year period  between April 1997 and March 2012 | Ciprofloxacin, norfloxacin, levofloxacin, moxifloxacin,  and ofloxacin (n=657 950) | Aortic aneurysm and  aortic rupture or dissection in hospital and emergency departments | Population-based longitudinal cohort study of elderly patients in Ontario, Canada | Adults with no prescriptions for fluoroquinolones (n=1 086 410) | 2 to 17 years | Demographics,  healthcare utilization and comorbidities | Use of any fluoroquinolone up to 30 days  following treatment | AA | 2.24, (2.02-2.49) |
|  |  |  |  |  |  |  |  |  | AD | 2.84, (2.32- 3.50) |

**Table 1C** Characteristics of Included Observational Studies Comparing Fluoroquinolone Use With Nonuse or Exposure to Other Antibiotics Regarding the Risk for Aortic Disease

| Study  Authors  Year | Participants | Interventions | Control | Primary  Outcomes | Study design | Follow-up | Covariate  adjustment | Exposure category | Outcomes | Adjusted OR (95% CI) |
| --- | --- | --- | --- | --- | --- | --- | --- | --- | --- | --- |
| Lee et al.  2018 | All inpatients diagnosed with AA or AD from  2000 to 2011 in longitudinal health insurance database | Ciprofloxacin,  levofloxacin, ofloxacin, sparfloxacin, norfloxacin,  lomefloxacin, moxifloxacin, gemifloxacin, enoxacin,  pefloxacin (N=1213) | DRS matching process among participants not exposed to fluoroquinolones (N=1213) | Aortic aneurysm  or aortic dissection | unidirectional case-crossover design | 60-180 days | A disease-risk score–matched time control analysis was performed to investigate the potential time-trend bias.  Risks were calculated by a conditional logistic regression model | Using the predefined 60-day window during  the case or reference period  a reimbursement code of oral fluoroquinolones with a prescription length of 3 days  or more. | Aortic Aneurysm or Aortic Dissection | 2.05,  (1.13-3.71) |

**Table 1D** Characteristics of Included Observational Studies Comparing Fluoroquinolone Use With Nonuse or Exposure to Other Antibiotics Regarding the Risk for Aortic Disease

| Study  Authors  Year | Participants | Interventions | Controls | Primary  outcomes | Study design | Follow-up | Covariate  adjustment | Exposure category | Outcomes | Adjusted ROR (95% CI) |
| --- | --- | --- | --- | --- | --- | --- | --- | --- | --- | --- |
| Sommet et al. 2019 | patients ≥  50 years diagnosed with AA or AD from 1972 to 2017 in the World Health  Organization Global Individual Case Safety Reports (ICSRs)  Database (N=172588) | Levofloxacin,  Ciprofloxacin,  Moxifloxacin,  Ofloxacin,  Gatifloxacin,  Tosufloxacin | Patients taking amoxicillin (N=40658) | Aortic aneurysm  or aortic dissection | Case/no-case | NR | Age, sex, year of report, continent of report, notifier type,  and number of drugs prescribed were analyzed by multivari-  able logistic regression | Exposure to fluoroquinolones  vs. amoxicillin exposure | AA or AD | 2.78, (1.83–4.23) |

**Table 1E** Characteristics of Included Observational Studies Comparing Fluoroquinolone Use With Nonuse or Exposure to Other Antibiotics Regarding the Risk for Aortic Disease

| Study  Authors  Year | Participants | Interventions | Controls | Primary  outcomes | Study design | Follow-up | Adjustments | Exposure category | Outcomes | Propensity scores  60-day risk period  HR (95% CI) | Propensity scores  61-  to 120-day risk period  HR (95% CI) |
| --- | --- | --- | --- | --- | --- | --- | --- | --- | --- | --- | --- |
| Pasternak et al.  2018 | Population included all adults in Sweden  who received a prescription for fluoroquinolones or  amoxicillin during the study period and who were aged 50  years or older from July 2006  to December 2013 | Fluoroquinolones exposure (360088） | Amoxicillin  Use (n=360088) | New-onset aortic aneurysm or dissection | A cohort study based on linked  nationwide data from Swedish registers | 120 days | Propensity scores | A 60-day period from start of treatment (days 1 to 60, starting from the date when the  prescription was filled).  A secondary  analysis investigating the subsequent 60 days (days 61  to 120) | Aortic aneurysm or dissection | 1.66; (1.12- 2.46) | Aortic aneurysm  1.90 (1.22 to 2.96) |
|  |  |  |  |  |  |  |  |  |  |  | Aortic dissection  0.93 (0.38 -2.29) |
