# Supplementary material for: Relationship between fluoroquinolones and the risk of aortic diseases: a meta-analysis of observational studies
Source: BMC Cardiovasc Disord. 2020 Feb 3;20:49. doi: 10.1186/s12872-020-01354-y (PMC6998374; doi:10.1186/s12872-020-01354-y)
Supplement: Supplementary file 2 — Additional file 2. Quality Assessment of Included Studies by Newcastle–Ottawa Scales. [file 12872_2020_1354_MOESM2_ESM.docx]

**Table 2** Risk of bias of observational studies reporting on aortic diseases using the Newcastle Ottawa Scale

| Lee et al. 2015 |  | NOS SCORES:★★★★★★★★ |
| --- | --- | --- |
| **Selection** |  |  |
|  | Adequate case definition | An independent validation had a positive predictive rate of 92%;  A more specific outcome definition by combining with surgical procedures; |
|  | Representativeness of cases | Representative cases |
|  | Selection of controls | Population-based |
|  | Definition of controls | One hundred controls were selected for each case, matched for 5-year age class, sex, and the index date of case diagnosis. |
| **Comparability** |  |  |
|  | Comparability of controls | Study controlled for all 96 covariates by using conditional logistic regression analysis.  A propensity score for adjustment and matching due to conventional multivariate analysis.  Cases have a higher burden of cardiovascular diseases, higher Charlson index, and greater use of cardiovascular medication than controls |
| **Exposure** |  |  |
|  | Ascertainment of exposure | Records of patients having a fluoroquinolone prescription |
|  | Same method for ascertainment  of cases and controls | YES |
|  | Nonresponse rate | Non-respondents described |
| Daneman et al.2015 |  | NOS SCORE:★★★★★★★★ |
| **Selection** |  |  |
|  | Representativeness of the exposed cohort | Truly representative of adults 65 years and older; drawn from a population-based Ontario Registered Persons Database. |
|  | Representativeness of the nonexposed cohort | Drawn from the same database |
|  | Ascertainment of exposure | Records of fluoroquinolone prescriptions for individuals in database. |
|  | Outcome of interest was not present at the beginning of study | Interest outcomes were identified at inception of this study |
| **Comparability** |  |  |
|  | Comparability of cohorts because of the design or analysis | Study controls for negative tracer and positive tracer |
| **Outcome** |  |  |
|  | Assessment of outcome | International Classification of Diseases 9th and 10th diagnoses |
|  | Follow-up long enough for outcomes to occur | A minimum of 2 years and a maximum of 17 years follow-up |
|  | Adequacy of follow up of cohorts | Complete |
| Pasternak et al.2018 |  | NOS SCORE:★★★★★★★★ |
|  |  |  |
| **Selection** |  |  |
|  | Representativeness of the exposed cohort | True representativeness; all adults in Sweden who use fluoroquinolones or amoxicillin during the study and who were aged 50 years or older in database (nationwide data from Swedish registers). |
|  | Representativeness of the nonexposed cohort | Drawn from the same database. |
|  | Ascertainment of exposure | A first diagnosis of aortic aneurysm or dissection (admission to hospital or emergency department for aortic aneurysm or dissection, or death due to aortic aneurysm or dissection) associated with oral fluoroquinolone use. |
|  | Outcome of interest was not present at the beginning of study | Interest outcomes were identified at the inception of this study |
| **Comparability** |  |  |
|  | Comparability of cohorts because of the design or analysis | Study controls for potential confounding from differences in baseline health status identified with propensity score matching.  Propensity scores using to control for potential confounders, treatment episodes of fluoroquinolone and amoxicillin use |
| **Outcome** |  |  |
|  | Assessment of outcome | Diagnoses according to ICD-10 (International Classification of Diseases, 10th  revision) and data for surgical procedures. |
|  | Follow-up long enough for outcomes to occur | 120-day follow up |
|  | Adequacy of follow up of cohorts | Complete |
| Sommet et al.2018 |  | NOS SCORE:★★★★★★★★ |
| **Selection** |  |  |
|  | Adequate case definition | Cases were ICSRs (the World Health Organization Global Individual Case Safety Reports) containing the term “aortic aneurysms and dissections” according to the MedDRA dictionary. |
|  | Representativeness of cases | Clearly representative series of cases |
|  | Selection of controls | Population-based |
|  | Definition of controls | All others except those with aortic aneurysms and dissections in the same database |
| **Comparability** |  |  |
|  | Comparability of controls | Adjusted for age, sex, year of report, continent of report, notifier type, and number of drugs prescribed; analyzed by multivariable logistic regression. |
| **Exposure** |  |  |
|  | Ascertainment of exposure | Fluoroquinolones and amoxicillin use recorded in national pharmacovigilance systems |
|  | Same method for ascertainment  of cases and controls | YES |
|  | Nonresponse rate | Non-respondents described |
| Lee et al.2018 |  | NOS SCORE:★★★★★★★★ |
|  |  |  |
| **Selection** |  |  |
|  | Adequate case definition | Cases were required to have International Classification of Diseases (ICD-9-CM) codes for  AA or AD, plus diagnostic evidence obtained by advanced imaging studies such as angiography, transesophageal or transthoracic echocardiography, thoracic or abdominal computed tomography, or magnetic resonance imaging. |
|  | Representativeness of cases | Representative cases |
|  | Selection of controls | Population based |
|  | Definition of controls | Self-controlled method based on case-crossover design instead of selecting controls from an external population |
| **Comparability** |  |  |
|  | Comparability of controls | Self-controlled method reduces the possibility of within-person time-invariant confounding, and avoids control selection biases.  Case-time-control design adjusts for the exposure-outcome association derived from case-crossover analyses. |
| **Exposure** |  |  |
|  | Ascertainment of exposure | Exposure to fluoroquinolone was identified by a reimbursement code of oral fluoroquinolones with a prescription length of 3 days or more. |
|  | Same method for ascertainment  of cases and controls | YES |
|  | Nonresponse rate | Non-respondents described |
